# Perspectives of transformative learning and professional agency: A native Chinese language teacher’s story of teacher identity transformation in Australia

**DOI:** 10.3389/fpsyg.2022.949673

**Published:** 2022-08-22

**Authors:** Suling Yang, Jinghe Han

**Affiliations:** ^1^School of International Studies, NingboTech University, Ningbo, China; ^2^School of Education, Western Sydney University, Penrith, NSW, Australia

**Keywords:** native CFL teachers, teacher identity, professional agency, Mezirow’s transformative learning theory, narrative inquiry

## Abstract

The notion of teacher identity has gained momentum in second language (L2) teacher education in the past decade. However, the research into Chinese as a Foreign Language (CFL) teacher identity has yet to receive more attention. The study employed a narrative inquiry to explore a native Chinese CFL in-service teacher’s identity negotiation and transformation within an international teacher education program. Self-reported narrative accounts, including multiple in-depth interviews and once-a-term reflective journals, were complemented by field notes and program documents. This data captured how the participant teacher negotiated internally with self and externally with the new environment to pursue professional growth. Mezirow’s transformative learning theory was used to reveal the cognitive trajectory of the participant’s teacher identity transformation with critical reflection as the central stage. Further, guided by Eteläpelto et al.’s framework of professional agency, the study also unraveled multiple external and internal influences on the transformational trajectory. The findings confirmed the value of integrating these two theoretical perspectives to explore language teacher identity development and offer insights into L2 teacher education practices focusing on teacher identity development.

## Introduction

Language teacher identity has been an ever-growing research focus in the field of L2 teacher education in the past decade (Fairley, 2020). Teacher identity development is acknowledged by some researchers as central to or even synonymous with language teacher education itself (Varghese et al., 2016; De Costa and Norton, 2017; Yazan, 2018; Fairley, 2020). The justifications for such arguments are that teacher learning involves cultivating a new identity rather than just learning new skills and knowledge of teaching (Clarke, 2008), and the acquisition of teacher self-knowledge is conceptualized as part of teacher identity development (Kanno and Stuart, 2011).

The significance of researching language teacher identity is also due to its analytical capacity. As a “research frame” (Olsen, 2008, p. 5) language teacher identity has been widely used in theorizing and examining L2 teacher learning and professional development in various contexts. It has also started to gain attention as a central construct used to examine the implementation of teacher identity as a “pedagogical tool” (Olsen, 2008, p. 5) and “pedagogical intervention” (Morgan and Clarke, 2011, p. 825) in language teacher education practices. For example, teacher educators have been reported examining a wide array of developed learning tools or pedagogical approaches by TESOL teachers in the development of student teachers’ identity such as digital storytelling, Reflective Teaching Model, strength-based mentoring (Yazan and Lindahl, 2020) and critical auto-ethnographic narrative (Yazan, 2018). From a conceptual perspective, Fairley (2020) proposed a model of ‘identity-centered’ language teacher education. Within this model, she argued that the goal of language teacher education is developing a language teacher identity that is “transformative, agentive, advocacy (TAA)-oriented” (p. 1040). Fairley (2020) further proposed a competencies-based approach as the foundation of the language teacher education model. The development of this model was based on the existing literature. This model has offered a conceptual direction for identity-oriented language teacher education practices within the field of L2.

Despite the significance of researching language teacher identity and the burgeoning of teacher identity literature in L2 over the past decade, studies of CFL teachers’ identity development have been scant (Moloney and Xu, 2015; Ballantyne, 2018). This is especially so in the context of Mainland China (Gong et al., 2020). The limited research body of CFL teacher identity has been mainly confined in cross-cultural settings to immigrant native CFL teachers (Wang and Du, 2014; Zhang, 2015); Confucius Institute assigned CFL teachers working in overseas countries (Ye, 2017; Zhang, 2018; Xiang, 2021); and native Chinese student teachers in international education programs (Ballantyne, 2018; Wang, 2022; Han and Ji, 2021). Amongst this research, one recurring theme is the negotiation of cultural values and cultural identity as an overall blanket factor for CFL teachers’ identity shift. Examining teacher identity through the lens of cultural values and cultural identity as such belied the complexity of the issue and failed to capture the much more dynamic and multilayered identity negotiation and transformation process.

For example, Wang and Du (2014) studied some immigrant CFL teachers in Denmark and revealed that these teachers experienced identity shifts from a parental and moralistic approach to that of learning facilitator and culture worker. This shift was found due to the teachers’ experiences of the differences between Chinese and Danish social and educational culture. Similarly, Li (2016) investigated CFL immigrant teachers’ experience in Western-based schools. That research found that the immigrant CFL teachers constructed successful professional identities through involving “an effective blend of Eastern and Western cultural values and pedagogical practices” (p. 177). In Zhang’s (2015) study, the construction of CFL teacher identity was found influenced by teachers’ mediation of values among varied cultural discourses and between their culture and other cultures. Whilst the above studies examined immigrant native CFL teachers’ experiences, Han and Ji (2021) concentrated on CFL student teachers’ professional identity construction through a Sino-Australian teacher education program. This research revealed that most of the participants chose to adapt to the Australian culture by moving away from their cultural self in the new teaching and learning environment. However, the role played by the teacher education program in this identity shift was not considered. These studies did not attempt to disclose the nuanced internal processes of identity shift.

Studies of CFL teachers’ experiences in cross-cultural contexts also attended to teacher agency in the process of teacher identity development. However, the literature tended to be more focused on CFL teachers’ internal resources, leaving unaddressed those external forces interacting with internal conditions, that impact on teacher agency. For example, in a study of Confucius Institute teachers’ identity, Xiang (2021) studied old-timer and newcomer teacher identities finding that the old-timer teachers negotiated their own meanings for teaching, whereas peripheral identity of the newcomers was likely to constrain their agency in making their own voices. In a very similar way, Ballantyne (2018) studied a group of CFL student teachers’ process of becoming a teacher, suggesting that this group’s professional agency developed from the interplay between the development of teaching skills, engagement in a professional learning network and self-confidence. This study indicates that the enactment of agency enabled teacher candidates to continue in their trajectory of learning and identity construction. The shaping power of external forces on teacher agency was to an extent diminished.

This present study is intended to enrich the research areas indicated above. It is a narrative inquiry aimed to examine how an in-service native CFL teacher, Qing by pseudonym, negotiated and transformed her teacher identity in a Sino-Australian teacher education program. Conducting this study is meaningful in two respects, theoretical and empirical. Theoretically it builds on perspectives of Mezirow’s (2000) transformative learning theory and professional agency (Eteläpelto et al., 2013). This study, framed under these two conceptual lenses, will extend the existing research of CFL teacher identity by providing deeper and more nuanced interpretations in terms of the cognitive processes of teacher identity transformation, as well as the achievement of teacher agency and the interplay between teacher agency and teacher identity transformation. Secondly, through exploring a native CFL teacher’s lived experience, the present study aims to contribute to the broad L2 research landscape of teacher identity and identity-oriented language teacher education practices.

To achieve the research aims of the study, the following research questions will be answered:

(1) How did the participant teacher negotiate and transform her teacher identity from the perspective of transformative learning?
(2) What individual and sociocultural factors constrained and/or resourced the participant teacher’s agency in teaching?
(3) How was the agency connected with the participant teacher’s transformative learning and teacher identity development?

## The theoretical framework

### Conceptualizing teacher identity

Teacher identity^1^ is defined as one’s self-understandings in relation to other people and wider social structures, which shift across time and space and concern one’s considerations of future possibilities (Norton, 2013; Barkhuizen, 2016). It is not a fixed product, but a continuum developed through social contacts (Burns and Bell, 2011). The development of a teacher’s professional identity is always dependent on employment that is shaped by institutional and political contexts (Burns and Bell, 2011). These definitions suggest a postmodern view of identity, articulating teacher identity as social, discontinuous and multiple (Gergen, 1991). Whilst the present study acknowledges the postmodern characterizations, it also concurs with Akkerman and Meijer (2011) that a totally de-centralized view of identity is not possible and there is a need to account for unity, continuity and individuality in identity as well. This is why this study adopts a narrative approach to conceptualize teacher identity as “self-as-teacher stories” (Ruohotie-Lyhty, 2013, p. 122).

This narrative definition of teacher identity understands teachers as active agents in striving to sustain a coherent and continuous understanding of self-experiences over time through shifting educational and social landscapes (Ruohotie-Lyhty, 2013). A life-story is an individual construction and the one who lives the life inevitably carries the responsibility of sustaining its narrative coherence of multiple meaningful experiences (Carr, 1986). Teachers reflect on and selectively integrate new experience into their self-stories (Beijaard et al., 2004). It is a “subjective achievement,” a process that necessitates some striving and choosing (Coldron and Smith, 1999, p. 715). In other words, they shape their histories and “restory” themselves with reference to personal intentions and abilities (McAlpine and Amundsen, 2009).

This study thus takes a multi-focus lens to conceptualize teacher identity: seeing teacher identity as individual, unified and continuous whilst still acknowledging the social, multiple and discontinuous characterizations. To capture the participant teacher’s lived experience of teacher identity transformation that reflects these multiple aspects, this study draws on Mezirow’s (1996, 2000) transformative learning theory and a subject-centered socio-cultural approach to professional agency (Eteläpelto et al., 2013). Mezirow’s theory, as a “broadly humanistic theory” (Hodge, 2014, p. 165), frames identity as an individual, unified and continuous construction (Kirschenbaum and Henderson, 1989, p. 14; Anderson, 2001; Kellner, 2003; Dirkx, 2007) and construction of identity as a rational process located in the individual (Clark and Wilson, 1991). Thus, Mezirow’s theory offers a lens to examine the cognitive and developmental trajectory of a teacher’s identity negotiation and transformation. However, Mezirow’s theoretical framework does not adequately address the role of social influences on the process of transformation (Erichsen, 2011). This also suggests that Mezirow does not offer conceptual tools to illuminate the way in which multiple selves interact in this process as “multiplicity arises from the interplay of … powerful social forces on the individual” (Clark and Dirkx, 2000, p. 115). For this reason, this study employs a subject-centered socio-cultural approach to professional agency (Eteläpelto et al., 2013) to interpret the participant teacher’s narrative accounts Mezirow’s theory fails to capture. This lens of agency sees the achievement of teacher agency as resulting from the interplay between individual and contextual resources and constraints (Tao and Gao, 2021). Further, as also argued, “the exercise of agency forms professional identity and establishes its maintenance and transformation” (Vähäsantanen, 2015, p. 10). It thus can be assumed that this lens will enable the exploration of how the multiple facets of the participant teacher’s professional self and socio-cultural contexts within which she was situated interact to inform her teacher identity transformation. The following section will illustrate these two theories and how they will be used in this study.

### Mezirow’s transformative learning theory

Mezirow (1996, 2000) describes transformative learning as a process that generates significant and irreversible changes in one’s meaning perspectives. A meaning perspective (also referred to as a frame of reference) is defined as “a personal paradigm for understanding ourselves and our relationships” (Mezirow, 1978, p. 101) and is “the results of ways of interpreting experience” (Mezirow, 2000, p. 16). As prior meanings constructed out of past experiences become problematic in construing and guiding how we feel, think, or act about new experiences, they become a catalyst for us to revise our ways of relating to the world and make them more true, inclusive and justifiable for guiding future actions (Mezirow, 1978, 1996). This thus means that transformative learning indicates a fundamental change in one’s sense of self (Dirkx, 2007).

The present study will utilize the five fundamental stages that make up Mezirow’s (2000) transformation model. The process of transformation, as theorized by Mezirow (2000), often starts with a “disorienting dilemma” where individuals are aware of a mismatch between their prior assumptions and the expectations and demands from new experiences. A disorienting dilemma is often a significant personal event (Taylor, 2000) that represents a seed of new awareness, and exploring this awareness indicates a possibility of transformation (Poutiatine and Conners, 2012). Overseas teaching experience, such as that of the participant teacher in this study, is often seen as giving rise to disorienting dilemmas (Jacobs and Haberlin, 2022). As a consequence of the disorienting dilemma, individuals are triggered to engage in critical reflection on their prior assumptions about themselves and the world as well as those of others. This means that a process of critically assessing one’s own assumptions is often accompanied by critically reflecting on those of others (Mezirow, 2000). For instance, in the present study, the participant teacher’s critical insight into her own teacher identity was inextricably intertwined with her critical reflection on the new educational context. Critical reflection can lead to the construction of a new framework that transforms individual ways of making meaning of experience in the world and is considered essential for transformative learning to occur (Mezirow, 1990; Taylor, 2017). Moreover, transformative learning involves individuals’ participation in “reflective discourse” (Mezirow, 2000, p. 11). They talk with others to achieve a better interpretation of an experience and search a common understanding. This then leads to the validation of new interpretations. Reflective discourse may include interactions within a group or between two persons (Mezirow, 2000). Action follows to live the new perspective, which is an imperative part of transformative learning (Baumgartner, 2001). Mezirow (2000) suggests that behavioral change may start with individuals trying to live out their emerging new perspectives through which they build competence and self-confidence in the perspectives. He also indicates that living the new perspective by integrating it into life is essential for transformative learning.

### A subject-centered socio-cultural approach to professional agency

The construction and development of a teacher’s professional identity requires considerations upon professional agency (also referred to as teacher agency) as identity development takes place through his or her activity (Beijaard et al., 2004; Ruohotie-Lyhty and Moate, 2016). When teachers consider why and how to act, they act on and sustain the negotiation of their professional identity (Duff, 2012). This has the potential to lead to identity development and transformation (Chaaban et al., 2021). Lai et al. (2016) argues that agency is required to drive the development of a teacher’s professional identities. Thus, it can be assumed that agency is a means whereby the negotiation and transformation of a teacher’s professional identity can be encountered and theorized.

The present study uses a subject-centered socio-cultural approach to begin to conceptualize professional agency. This approach is put forward by Eteläpelto et al. (2013). Within this framework, professional agency is “exercised when professional subjects and/or communities influence, make choices, and take stances on their work and professional identities” (p. 61). Professional agency is negotiated and achieved through a relational interaction between the individual and the social, thus, functioning as “a pathway between social determinism and highly individualistic accounts of cognition” (p. 56). This means, on the one hand, individuals’ professional identities, work experiences, knowledge, and competencies function as developmental resources for their professional agency. On the other hand, professional agency is always exercised within specific contexts, locales or environments and these external conditions (e.g. power relations, work cultures and discourses) serve as constraints or resources. This framework entails a temporal dimension, emphasized by Priestley et al. (2015) in describing teacher agency as achieved within “a configuration of influences from the past, orientations toward the future and engagement with the present” (p. 23). This indicates that all the three dimensions are involved in teachers’ concrete agentic choices and actions, but the degree to which they contribute varies in each and every instance of agency achieved (Priestley et al., 2015). This also suggests that the practice of agency is dynamic.

The conception of professional agency as discussed above is conceptualized as having two components: one being “identity-agency” (Eteläpelto et al., 2015, p. 665), and the other involving an individual’s participation in one’s work and the work community (Ruohotie-Lyhty and Moate, 2016). Identity-agency is enacted when teachers renegotiate the components of their professional identity such as ideals, interests and goals, in interaction with social suggestions (Eteläpelto et al., 2015). This study is interested in unraveling the participant teacher’s participation in her work and the school community. Specifically, this study’s focus in terms of agency is on which areas of work and community the participant participated in and to what degree she participated. Agency in terms of teachers’ participation in their work and work community does not directly relate to their professional identity, but it constitutes a resource for them to draw on as they practice identity-agency (Ruohotie-Lyhty and Moate, 2016). This suggests that this form of agency is also involved in identity development. To put it differently, as teachers enact agency at work through choosing problems to engage in and with different degrees of engagement, they are defining what is learned or changed through their engagement (Billett, 2006). In this way, participation and learning are related to teachers’ subjectivities and identities (Eteläpelto et al., 2013).

A teacher’s internal forces interact with external forces (e.g., power relations, work cultures, and discourses) in shaping his or her teacher agency (Eteläpelto et al., 2013). Regarding internal forces, this study pays attention to the participant’s teacher identity and researcher identity. A university teacher’s professional self can be seen as made up of these two identities as his or her work generally relates to teaching and research activities based on subjects (Clarke et al., 2013). The participant teacher was still affiliated with a Chinese university whilst pursuing a doctoral degree in Australia. Moreover, teacher identity can have multiple facets, or sub-identities (Akkerman and Meijer, 2011), as well as imagined identities (Kanno and Norton, 2003). This study assumes the interplay between external forces and the participant’s multiple identities in the shaping of her teacher agency.

## Teacher narratives and restorying

Methodologically, this study was approached through narrative inquiry, a means to capture lived experience. Bruner (1987) proposes the narrative mode of knowing, arguing that we live in a storied world and narrative is the central structure of making meaning of experience. Connelly and Clandinin (2006) see story as “a portal through which a person enters the world and by which their experience of the world is interpreted and made personally meaningful” (p. 479). Empirical studies on teachers’ stories through the method of narrative inquiry has substantially enriched our understanding of teacher identity (Ottesen, 2014) and has also demonstrated its advantage in capturing teachers’ personal stories for exploring their professional identity and agency (Chaaban et al., 2021).

In narrative inquiry, participants tell stories of their experiences and researchers describe such experiences, collecting and retelling participant stories for research purposes (Connelly and Clandinin, 1990). In this “restorying” process, researchers are agents as they endeavor to draw out stories from participants and analyze raw data to form new stories for particular audiences (Mulholland and Wallace, 2003; Beaven and Jerrard, 2012). It is a process in which the original narrative of the participant is shared with the researcher, and interpreted and re-interpreted during the collaboration between the researcher and the participant.

### The context of the narrative inquiry and the participant

The narrative inquiry is situated in a Sino-Australian Language Teacher Education Program. Every year, volunteer-teachers from China with varying professional and educational experiences were recruited to engage in the school-based and workshop-facilitated professional learning, supporting or providing Chinese lessons in Australian classrooms. They were concurrently trained as researchers, enabling them to reflect on their professional learning. The program was aimed to prepare language teachers with international vision and inclusive pedagogical knowledge for language education. The participant teacher, Qing by pseudonym, joined the program as a volunteer-teacher and a doctoral student in September 2019. The first author of the article was also a volunteer-teacher and a doctoral student in the program. The study reported in this article is a part of the first author’s doctoral research project.

### Collecting narrative accounts

In this study, the primary sources of data include in-depth interviews and the participant’s three termly reflective journals. The self-reported original narratives are complemented with the first author’s field notes and the documents of the program. Methods employed for this study seem to be in line with those in other similar studies. For example, Kayi-Aydar (2019) conducted a single-case narrative study on a language teacher’s agency in the development of her professional identities by including data sources of interviews and journal entries of a course. Firstly, data collection began with the participant teacher’s three termly reflective journals. Qing, as a volunteer-teacher of the program, was required to write a reflective journal for each term in English as part of her course. The journals served as a powerful lens to enter Qing’s inner world as she recorded and reflected on major events she had experienced and negotiated in Australia. Then, three in-depth interviews were conducted and audio-recorded. The first two interviews revolved around Qing’s learning and teaching experiences both in China and in Australia. The focus was on her professional identity experiences in Australia. The third interview was conducted to capture more nuances on interesting points that had emerged in the prior interviews and reflective journals. The total length of interviews was 4 h. In addition, with Qing’s permission, fragmented and informal communication between the first author and the participant were also collected in the form of field notes. As Chinese volunteer-teachers and doctoral candidates in the Sino-Australian program, they often shared stories of teaching and learning in Australia both in person and *via* WeChat. The field notes also included ongoing member-checking conversations, which will be detailed later in this section. Finally, relevant documents of the Sino-Australian program’s background and policies were collected. These documents provided valuable information for facilitating the interpreting of Qing’s lived experiences.

### Analyzing and re-organizing the story

Data analysis of the study included three stages. The first and second stages were coding for broad themes to form a thematic map (Braun and Clarke, 2006). In the third stage, following the thematic map, the raw data was reorganized into a story that highlighted the meanings of Qing’s lived experiences.

The coding method employed in this study was similar to Braun and Clarke’s (2006) “theoretical thematic analysis” in which coding is more “driven by the researcher’s theoretical or analytic interest in the area” (p. 84). It was also informed by the assumption that theoretical perspectives enable narrative inquirers to gain further insights into narrative accounts (Clandinin and Connelly, 2000). The first stage was coding for identifying the salient meaning perspectives that built Qing’s professional identity constructed in China. The second stage was guided by the five fundamental stages that make up Mezirow’s (2000) transformation model and a subject-centered socio-cultural approach to professional agency (Eteläpelto et al., 2013). It was aimed at generating themes that, respectively, indicated: (1) the phases of the cognitive developmental trajectory of identity transformation; (2) the internal and external factors that interacted to shape Qing’s participation in teaching work and the school community.

To obtain narrative interpretations, Clandinin and Connelly’s (1994, 2000, 2006) “three dimensional” space was considered. The narrative structure consists of “temporality,” “sociality,” and “place.” By “temporality,” Qing’s story was traced back to the past, situated in the present, and pointing to the anticipated future. By “Sociality,” consideration was given to Qing’s relations to self and other people. By “place,” Qing’s story unfolded in China and Australia, two different contexts. Thus, this narrative structure provides space for addressing the spatiotemporal, personal and social dimensions of the participant’s lived experience.

### The researcher’s positionality and trustworthiness

This study falls into the category of “insider research” since the first author and the participant were both Chinese volunteer-teachers in the Sino-Australian program (Kanuha, 2000). Insider research is frequently subject to concerns of legitimation and trustworthiness. For example, researchers’ familiarity with the research context and participants can result in loss of objectivity in data interpretation (Asselin, 2003; Unluer, 2012). Great familiarity can also lead researchers not to probe for deeper meanings of the phenomenon under study or ignore crucial data in data collection (Asselin, 2003; Tshuma, 2021). Due to these potential pitfalls, insider researchers run the risk of compromising the trustworthiness of the study.

In response to this concern, an exhaustive and ongoing member-checking technique (Poole, 2019) was applied. This means that the interview transcriptions and field notes were returned to the participant for checking, and the emerging story was also repeatedly shared with the participant. Follow-up conversations were organized to obtain new information from the participant and negotiate alternative insights and interpretations. Thus, the interpretations reported in this study were co-constructed between the first author and the participant throughout the development of the “restorying” process (Ollerenshaw and Creswell, 2002). This process lasted for over 2 years and involved nuanced and iterative renegotiations and reinterpretations of the stories between the two sides. It ensured collection of high-quality data and sufficient probing of the participant’s meanings.

## Results – Qing’s story of teacher identity transformation

The results of this study are organized into a story of Qing’s teacher identity transformation. This story consists of four sections that present the background of her story, the cognitive developmental trajectory of her teacher identity transformation, the individual and sociocultural influences that contributed to the achievement of her agency in teaching, and how the agency was connected with her teacher identity development.

### The background of Qing’s story

Prior to attending the teacher education program in Australia, Qing had taught CFL at a Chinese university for almost 7 years. She described herself as a “professional teacher of CFL” (Qing: interview, October 23, 2020) and this self-perception comprised two main themes: a language trainer who thought of systematic Chinese linguistic acquisition as central to CFL education, and an expert teacher in this field. She also identified herself as a confident and responsible teacher. As Qing entered the Sino-Australian program as a Chinese volunteer-teacher in 2019, her prior teacher identity – the way in which she saw and valued her contribution to education – as a language trainer was challenged. This caused her to reflect upon and reconsider how she saw and valued herself as a consequence of both her past and present experience. This internalization of self-experience, however, was also not independent from the external influences of the new education landscape, one shaped by the teacher education program and the local school, nor was it free from influences of the multiple aspects of her professional self.

### Qing’s teacher identity transformation from Mezirow’s theoretical lens

Qing experienced a five-stage transformative learning trajectory during which her prior teacher identity as a language trainer was challenged, negotiated and transformed: (1) a disorienting dilemma; (2) critical reflection on her prior assumptions in relation to the new context; (3) reflective discourse; (4) trialing and further validating the new meaning perspective; (5) living the new perspective—integrating it into work.

#### A disorienting dilemma

Qing was allocated to a public primary school in Western Sydney – R Public School. There, the Chinese language program was run through the teacher education program and the school itself did not employ full-time teachers for CFL education. Qing, as a new volunteer-teacher, was required to observe the lessons of her volunteer-teacher colleagues who had taught at the school for a period of time.

The observation threw Qing into a “disorienting dilemma” (Mezirow, 2000). Central to their lessons was engaging students instead of helping students acquire the language. This, in her view, was unprofessional and irresponsible. This experience challenged Qing’s heretofore invisible and unquestioned assumption that a CFL teacher was a language trainer (Taylor and Elias, 2012). Initially, she rejected it altogether. She recalled:

I despaired. … My professionalism did not allow me to accept such Chinese lessons, and my sense of responsibility as a professional teacher did not allow me to teach like that. I hated to see an entire Chinese lesson spent in drawing a picture or learning to sing a song, with students learning nothing [not learning any Chinese language]. (Qing: Interview, October 23, 2020).

#### Critical reflection — “A language trainer” or “a teacher of interesting classes”

The disorienting experience triggered Qing to critically reassess her “own orientation to perceiving, knowing, believing, feeling, and acting” (Mezirow, 1990, p. 13) about CFL education and what she could or should do as a CFL teacher in the new context.

Qing began to assess the curriculum of CFL education at R Public in relation to her prior experiences. She became aware that systematic language acquisition was not stipulated as central to the language education she was expected to provide. Each class had only one Chinese lesson every week and each lesson lasted for only 30 min. For her, this limited teaching time, plus the minimal previous exposure to Chinese language and culture of her students, made systematic grammatical teaching in class practically impossible. Moreover, the CFL program at the school did not include learning outcome and assessment. Qing recalled: “The criteria for a successful Chinese lesson was whether students could discern Chinese characters from other languages [Japanese, Korean] [and] whether students enjoyed the lesson or engaged in classroom activities” (Qing: Interview, October 23, 2020). Very soon during the observational period, critical reflection on her prior teacher identity in relation to the new context led Qing to become aware that in this school a Chinese volunteer-teacher was seen as needing to be a “teacher of interesting classes.” She was expected to spark students’ interest in the language and its culture through the construction of enjoyable activities.

However, a fundamental change in her approach to CFL education did not occur until she began to develop a deeper understanding of the Australian students and their learning needs and cognitive abilities, and this in turn deepened her critical reflection into what was suitable CFL education for them. This took place when she had engaged in this school for about half a year. She stated: “I gradually accepted the interest-oriented approach to CFL education in Australia. I stopped sticking to grammar in the later [stages]… of my teaching experience in Australia.” (Qing: interview, August 22, 2021).

#### Reflective discourse — using professional dialogs to make sense of experience

Amid disorientation, Qing sought to have dialogs with her peer volunteer-teachers in the teacher education program and her friends teaching CFL in New Zealand and the United States. She also consulted with the coordinator and lecturer of the program. These dialogs revolved around the diverse contexts in which Chinese teaching took place. Through these discussions Qing compared and contrasted the contexts and critically reflected on the potential appropriate CFL pedagogies. This led her to learn more about her new experience and become surer that she had to renegotiate her prior teacher identity as a language trainer, “[so] I had to adapt and change [in response to the new environment],” in her own words. She narrated:

They (My volunteer-teacher colleagues) said that the Chinese program was not aimed at helping students to acquire the language, but promoting students’ interest in the language. … They all advised me not to stick to teaching students linguistic knowledge. (Qing: Interview, October 23, 2020).

Thus, in Qing’s story of transformative learning, rational discourse became an arena wherein her experience and critical reflection were played out (Taylor, 2000). Through these dialogs, she gained better understanding of the meaning of her new experience, sought for common understandings, and validated her changing interpretations of her new experience (Mezirow, 2000). In other words, Qing drew on collective experience to question her prior experience and promote her understanding of the new experience, and hence achieve a “tentative best judgment” (Mezirow, 2000, p. 11).

#### Trialing and further validating the new meaning perspective

Putting the new meaning perspective into practice is necessary for effecting transformative learning (Baumgartner, 2001). Qing’s emerging new meaning perspective led to major changes in her teaching practice in Australia. She explored pedagogies to spark students’ interest in learning Chinese. She said:

As a language trainer in China, my focus was on how to teach grammar effectively. … In the new context, however, I began to assess students’ conditions, to consider their needs, the things that would engage them, and even what they were learning in other subjects. (Qing: interview, August 22, 2021).

In an interview, Qing mentioned about how she amended teaching to engage students during the third term of 2020. She taught lessons based on what her students were learning in their mathematics class. Such lessons not only engaged students but also aroused local classroom teachers’ interest in Qing’s teaching and won their support. This further validated the new meaning perspective. She wrote: “I gained confidence when I was delivering lessons that were relevant to their course [Mathematics]. Classroom teachers also showed their interest in my lesson.” (Qing: Termly Reflective Journal, Term 3, 2020).

#### Living the new perspective — integrating it into work

Throughout almost a year’s professional learning in Australia, Qing’s conception of the nature of CFL education “expanded.” She realized that her prior understanding was limited and that depending on contexts, interest-sparking pedagogy should be included in the teaching practice of CFL teachers. Thus, Qing’s understanding of her teaching self became “more inclusive, discriminating, open,” and “more true or justified to guide action” (Mezirow, 2000, p. 7). It became more flexible and negotiable. She stated that she would utilize this new learning perspective in her future career, after she returned to her teaching position in China. After this occurred when the third interview was conducted, she said:

Now I am back in China and when I teach international students at university I strictly follow the principle of systematic linguistic acquisition. But when I need to provide Chinese lessons to students overseas, in particular if these students are young and the length of lessons is short, I think about what topics and what content would engage them and strengthen their interest in learning Chinese language and culture. (Qing: interview, August 22, 2021).

### Individual and sociocultural factors shaping Qing’s teacher agency

In Qing’s story, sociocultural and individual factors interacted shaping the achievement of her teacher agency (Eteläpelto et al., 2013). The sociocultural factors included the structural conditions and school culture within which she was embedded in Australia. The individual factors primarily referred to her multiple professional selves that were linked with her prior work and life history. These internal and external forces were inextricably intertwined in Qing’s attempts to navigate various conflicts and tensions and shaped her agentic choices and actions in terms of her participation in teaching and the school community.

#### Constraints of Qing’s teacher agency

Qing’s professional learning and teacher identity development in Australia was a story imbued by the constraints of structural power on her agentic choices and actions in relation to teaching and the school community. These structural factors included structural labeling that undervalued her expertise, a heavy teaching load and unequal power relations. In addition, tension between her teacher identity and researcher identity was also intertwined with these external forces.

Qing felt demotivated due to her being labeled a volunteer-teacher. Volunteer-teachers were identified as ‘unqualified teachers’ in the Sino-Australian program. They were, in the program coordinator’s words, “not supposed to be teaching, but to be learning to teach from the Australian teachers in the Australian system.” (Qing: Interview, December 19, 2020). This institutional discourse, in Qing’s view, was “ridiculous” and suppressed her initial enthusiasm for teaching at the school. As a CFL teacher, she used Chinese as her first language, was trained and received qualified teaching degrees in China, and had years of teaching CFL experience to international students in China. She acknowledged that she was learning from the local teachers. But, at the same time, she also thought that her expertise as a professional CFL teacher should be valued. However, the structural labeling of volunteer-teacher as ‘unqualified’ made her feel belittled and thus curtailed her engagement in her teaching work. In this regard, the interplay between her self-perception as a professional teacher constructed during her prior experience and the structural condition within which she was situated in this teacher education program informed the shaping of her teacher agency.

I expected to be seen as a professional teacher. I had hoped to provide good service to the program, and to help build a more effective CFL program at the school where I volunteered. But it appeared they [the coordinator of the program and the school mentor] did not accept it. So I thought I shouldn’t necessarily be as devoted as I had thought I should. … [I had those bad moments of telling myself:] just be there and finish the teaching duty. (Qing: Interview, December 19, 2020).

The heavy teaching load and unequal power relations involved also emerged as significant structural factors that constrained Qing’s teacher agency. These negative themes recurred in her narrative accounts of her experience before the teaching load was reduced at the third term of 2020. After the observational period ended and her volunteer colleagues left the school, Qing was required to teach the entire school. This meant that she had to teach twenty-one lessons over two consecutive days weekly. This, for her, was a difficult mission. Each lesson lasted 30 min and she taught 10 or 11 lessons daily. Her teaching started at 9:00 in the morning and ended at 3:00 in the afternoon. Except for a brief lunchtime break, she kept “hopping” non-stop from one classroom to another. Such intensity seemed to be one reason for her to frequently feel exhausted at the end of the teaching day. Moreover, she was a responsible teacher and lesson preparation required a great deal of time and energy for the wide range of student population from kindergarten to grade six. She said, “lessons for different grades had to be prepared and taught differently given their varying cognitive capabilities.” (Qing: Interview, October 23, 2020). Further, she was also a doctoral student constantly pressed for advancing research work. She frequently talked about suffering burnout after 2 days’ teaching and had to sleep throughout the following day, which affected her doctoral studies.

Qing mentioned about her attempts to bring her concern to the School’s management for adjustment of this unreasonable arrangement in interviews as well as in her reflective journal. She expected to be able to ensure teaching quality as well as research work with a more reasonable workload. She said, “I wanted to ensure teaching quality. I wouldn’t possibly have the time to know the students well and prepare lessons well enough when I had to take charge of so many classes.” (Qing: Interview, October 23, 2020). However, to her disappointment, there was no adequate response to her concerns. Feeling herself to be burdened with an overly heavy teaching load, and lacking institutional support, Qing felt “powerless” and did not have “any autonomy.” (Qing: Interview, December 19, 2020). This decreased her investment for teaching. She recalled: “Since they treated me like that, I got out there immediately after finishing teaching every time.” (Qing: Interview, December 19, 2020). Qing’s narration concerning the non-negotiable heavy teaching task displayed an inextricable interplay between structural forces and her individual conditions. Specifically, interacting with her ethical pursuit of responsibility, the heavy teaching load and unequal power relation together with her aspiration of doctoral degree appeared to be constraining her participation in teaching.

Qing’s participation in teaching was also hindered by her individual condition—her pursuit of doctoral studies. As mentioned in the preceding narration, there was tension between her teaching and doctoral studies, and this tension became particularly intense when she had to conduct a heavy teaching load and meanwhile her supervisor did not support her research work well. In interviews, she repeatedly talked about how the struggle to balance between the two became particularly intense as she was burdened with an unreasonably heavy teaching load. She apparently prioritized research training rather than teaching as she stated, “I was here [in Australia] primarily to finish my doctoral degree.” (Qing: Interview, October 23, 2020). It was essentially due to her strong aspiration for her future professional self—maintaining job security and earning a promotion in academia. As she said in one of the WeChat member-checking conversations: “As a university teacher, I have been facing ever-increasing pressure for not holding a doctoral degree. … A doctoral degree will certainly boost my capabilities and possibilities of achieving a higher professional title.” (Qing: field notes, December 19, 2021). Thus, Qing chose to reduce her investment in teaching for ensuring her commitment to becoming an academic researcher. She chose to “just get through teaching duties, … focus on [my doctoral] studies, don’t be concerned about teaching, don’t let teaching interfere with [my] mood and affect [my] doctoral studies.” (Qing: Interview, October 23, 2020). It was worth noting, however, that despite Qing valuing research training more than teaching and the priority seeming ‘clear-cut,’ the negotiation was visibly continuous.

#### Resources of Qing’s teacher agency: Individual, cultural, and structural

Situated within the structural constraints and constantly negotiating the distractions around dedication to her doctoral studies, Qing felt that her identity as a responsible teacher enabled her to sustain a certain sense of teacher agency. This individual resource of agency ensured her continuous search for suitable Chinese teaching for her Australian students. She ascribed this resource to her past professional experiences.

My past professional experience made me feel the responsibility as a teacher. That was, completing the allocated teaching task at the school as well as maintaining the [basic] quality of my teaching. I tried my best to engage my students and increase their interest in learning Chinese in my lessons. … I took providing good teaching as my duty. I did what a teacher should do … [I set out] to ensure the effectiveness of my teaching, and [to] do it well, which was my duty. (Qing: Interview, October 23, 2020).

Moreover, school culture also acted as a resource of Qing’s teacher agency (Eteläpelto et al., 2015). Local teachers were very friendly to her and this warm work climate and social relationship boosted a sense of belonging in her and thus motivated her to uphold a serious attitude to teaching at the school (Hökkä et al., 2017). She stated:

Classroom teachers would express their concern about my family back in China. They were all very friendly to me. This made me feel I was a colleague of theirs, … and I shouldn’t treat teaching there in a slipshod manner. (Qing: Interview, October 23, 2020).

Notably, Qing’s agency in teaching was also significantly enhanced due to a substantial cut in her teaching load. This occurred as a result of the school’s policy shift at the third term of 2020 when she had been teaching there for approximately half a year. Because of shortened weekly teaching hours, she was able to distribute more of her time and energy to exploring suitable Chinese lessons for the benefit of her Australian students. The dramatic change in Qing’s teacher agency due to a cut in the assigned teaching load demonstrated the power of institutional structuring in mediating a teacher’s agentic choices and actions in teaching practice.

### Connecting Qing’s teacher agency with teacher identity development

Teacher agency is enacted when a teacher performs concrete agentic choices and actions that affect his or her teaching practice (Tao and Gao, 2021) and it can be viewed as “the performance of identity within the constraints of teachers’ professional contexts” (Hiver and Whitehead, 2018, p. 3). Whilst the primary focus of the foregoing section is on how the internal and external factors shaped Qing’s participation in teaching, this following section will illuminate how her enactment of agency was associated with her professional learning and teacher identity development.

Prior to the third term of 2020, it appeared that Qing sustained a certain sense of teacher agency that was very similar to a “satisficing form of agency” (Chaaban et al., 2021, p. 6). This meant that she coped with the teaching duty in a way that she felt that her ethical standard as a responsible teacher was more or less maintained and the requirements of the teaching situation were met. This sense of agency was manifested in her agentic participation in classroom teaching. She would spend a whole day preparing her teaching every week and she made efforts in preparing differentiated instruction for different cohorts of students. Moreover, she said that “lifeless instruction [that might work for her adult students in China] was not for children,” and she “mobilized her facial expressions, language and tones as well as organized different classroom activities to engage the students.” Even though organizing and conducting classroom activities for children was “exhausting” and “different from that for her adult learners in China,” and that she was “suffering a sore throat” as a result of continuous enthusiastic teaching, she still tried to do what would be good for students. (Qing: Interview, December 19, 2020). These agentic actions provided a resource for Qing’s professional learning and teacher identity negotiation: she explored suitable ways of teaching for the Australian children and negotiating between her prior teacher identity and her emerging new teaching self in Australia.

Notably, at the third term of 2020 Qing’s participation in teaching was distinctly enhanced due to this reduction of her teaching load. As a responsible teacher, she took as her duty the effort to provide effective teaching. When she had to teach only 4 lessons for one teaching day, she was able to spend more time and energy in exploring the children’s needs and trialing an interest-oriented pedagogy. She narrated:

I was assigned 8 lessons for two consecutive days every week [for the third term of 2020]. Thus, I had more time and energy to spend on lesson preparation and in knowing more about the students. When I was waiting for my next lesson, I would take advantage of the time to observe classroom teachers’ lessons, … [and I was interested in] the content of math lessons and the topics of writing course. (Qing: Interview, October 23, 2020).

In her reflective journal, she also talked about her interest in students’ math class and used the content of the math lessons as her teaching material. She was motivated to “make the lessons creative, interesting, and future-focused” (Qing: Termly Reflective Journal, Term 3, 2020). These agentic choices and actions constituted a critical resource for her to achieve identity transformation: she attempted to live out her emerging new understanding of CFL education as “interest-oriented” and thus working toward developing an expanded teacher identity. This was considered as an essential process—behavioral change in Qing’s transformative learning and identity transformation, as illustrated in Section “Qing’s teacher identity transformation from Mezirow’s theoretical lens.”

On the other hand, her initial passion for participation in the school community outside the classroom was significantly suppressed. An excellent example of this was that at the outset of her school engagement, she tried “to be more involved in the school, … greet [local] teachers and try to find interesting topics with them” to know more about the school and students. (Qing: Termly Reflective Journal, Term 4, 2019). However, when talking about her experience during the first term of 2020, she described herself as a teacher who “only wanted to flee the school” and “left as soon as the teaching was done.” (Qing: Interview, December 19, 2020). This seemed indicative of her withdrawal from the school community. In addition, as a professional CFL teacher, she initially intended to work out a systematic CFL syllabus for the school. This plan was also abandoned due to her restricted agency. Qing’s non-participation suggested a loss of resources for her professional learning and teacher identity development (Vähäsantanen, 2015; Schutz et al., 2018).

## Discussion

**FIGURE 1:**
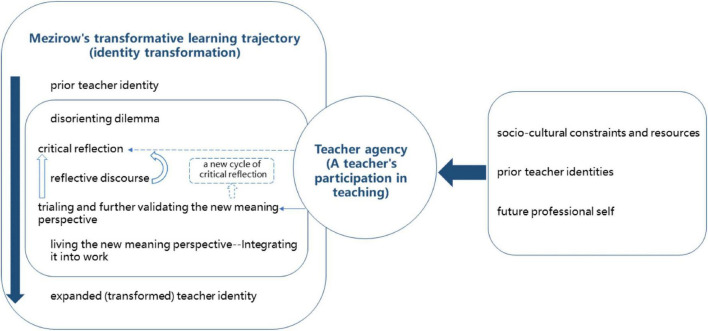
The cognitive trajectory of Qing’s teacher identity transformation, achievement of her teacher agency and connection between these two processes.

The teacher’s story corroborates the attitude that international teaching experiences can be seen as a form of disorienting dilemma (Mezirow, 2000) that can function as a catalyst for teachers to achieve transformative insights into their professional self (Ye and Edwards, 2018; Jacobs and Haberlin, 2022). Building on the results of this study, the subsequent discussion will revolve around four aspects (also see Figure 1) that contribute to the scholarly literature on transformative learning, teacher agency and L2 teacher identity development.

First, the teacher’s story demonstrated that critical reflection was the engine that drove her fundamental change and development. Mezirow (2011) argues: “Transformative learning may be understood as the epistemology of how adults learn to reason for themselves—advance and assess reasons for making a judgment” (p. 23). Through critical reflection, the teacher’s prior perceptions of CFL education and mission as a CFL teacher was deconstructed and interrogated (Mezirow, 1990). This opened up new meaning perspectives that were more “inclusive, differentiating, permeable, critically reflective, and integrative of experience” (Mezirow, 1996, p. 163). Specifically, evaluating the local Chinese language curriculum and the students at the school prompted and advanced her critical reflection on her prior assumptions, and her increasingly expanded knowledge of the students was decisive in moving her toward fundamental changes in attitudes and practices. The teacher’s critical self-reflection was also promoted through reflective dialogs (Mezirow, 2000) with other CFL teachers and the program’s teacher educator. This meant that these people’s experiences and ways of meaning-making were capitalized on as resources for facilitating her critical reflection and validating her new emerging insight (Mezirow, 2000). Further, based on her emerging meaning perspective, the teacher implemented a revised pedagogy for classroom teaching, reflected on and analyzed its effect on student learning. This consolidated her new insight due to positive feedback from students and classroom teachers. This seemed to be triggering a new round of critical reflection on “future-focused” pedagogy.

The teacher’s experiences echoed Liu’s (2020) argument that “critical reflection is a hermeneutic approach that involves repeated reexamination of one’s assumptions about knowledge and understanding” (p. 131). Critical reflection leading to transformative learning involves behavioral change and reflection on this change, which may also result in a new cycle of critical reflection (Liu, 2015). This study adds to the conceptualization of the hermeneutic approach by demonstrating that this process may be extended, particularly for experienced in-service teachers. Also, previous studies have reported the facilitative role of reflective practice in the identity development of teacher educators (Song, 2021) and ESL teachers (Farrell, 2013) as well as the intertwined relationship between teacher candidates’ reflective learning and identity construction (Yuan and Mak, 2018). Studies of CFL teachers have also had a mention of critical reflection as a way for negotiation of cultural values and cultural identity (Li, 2016). This study thus enriches the literature by revealing the dynamic and reciprocal relationship between critical reflection and behavioral change in the development of an in-service CFL teacher’s identity transformation.

Secondly, the findings are broadly aligned with those of previous studies which have shown that teacher agency is the outcome of the inextricable and complex interplay between individual and sociocultural resources and constraints (Vähäsantanen, 2015; Lai et al., 2016; Tao and Gao, 2017, 2021; Wang et al., 2021; Sun and Cheng, 2022). Structural and cultural factors conditioned the extent to which the teacher was able to translate intentionality into practice (Hökkä et al., 2012; Priestley et al., 2015). These factors included teaching loads, institutional discourses, unequal power relations and school culture. The story indicates that structural changes may increase the degree to which a teacher is able to act on intentionality and thus create potentiality for enactment of enhanced agency (Hinostroza, 2020). Moreover, the different facets of the teacher’s professional self emerged as individual resources and constraints in the achievement of teacher agency. The narrative presented an entanglement of teacher identity, researcher identity and structural issues. It showed how this tension informed the shaping of teacher agency. The complexity of interplay between a L2 teacher’s multiple identities in the shaping of agency within structural constraints of a cross-border teacher education program has not captured scholars’ attention, even though teacher-researcher identity tension is not new in studies of teacher educators’ agency (Hökkä et al., 2012; Hinostroza, 2020) and university academics’ identity conflicts (McCune, 2021). Further, the teacher’s story lent empirical support to the temporal dimension of teacher agency, “the dynamic interplay between past experience, engagement with the present and orientations toward the future” (Eteläpelto et al., 2013). The teacher’s prior teacher identity and her aspired future self encountered in the shaping of her teacher agency within the cross-border program.

The third aspect of the findings worth being highlighted is that teacher agency appears to be a site where teacher identity is negotiated or transformed. The teacher’s participation in classroom teaching (i.e., teacher agency) resourced her teacher identity development (Duff, 2012; Hökkä et al., 2012; Schutz et al., 2018; Chaaban et al., 2021). The findings indicate that a teacher’s agency is a potential resource for the process of critical reflection, the main internal drive for teacher identity transformation. Critical reflection may occur when a teacher attempts to solve problems and reflects on her or his own beliefs (Jacobs and Haberlin, 2022). Agency might resource a teacher to question and negotiate the prior teaching self, thus facilitating identity re-negotiation. Moreover, an increased sense of teacher agency can facilitate a teacher’s fundamental change in pedagogy. Previous research found that activities and practices teachers partake in and tools and resources they use are vital elements for teachers’ learning community (Yazan, 2018, p. 30). In this case, the time and effort the teacher spent on knowing students and attending local teachers’ lessons resourced her to implement an interest-oriented pedagogy, which further validated her emerging revised insight and was central to her transformation.

Finally, building on the discussion above, the teacher’s story has exemplified a holistic conceptual framework for describing and interpreting L2 teachers’ identity negotiation and transformation. The nuanced process of the teacher’s critical reflection demonstrates the conceptual strength of Mezirow’s theory in disclosing the internal rational process of teacher identity transformation. The power of Mezirow’s theory is also manifested in the conceptual space it provides for describing both structural changes in awareness and enactment of the transformed awareness (Baumgartner, 2001) that are both considered essential for teacher growth at an identity level (Meijer et al., 2017). On the other hand, a subject-centered socio-culturally based perspective of professional agency (Eteläpelto et al., 2013) captures the teacher’s narrative accounts concerning multiplicity of her professional self and the socio-cultural context where her personal transformation occurred, which Mezirow’s theory lacks. This latter conceptual lens allows the examination of the dynamic interplay between one’s individual conditions (e.g., multiple conceptions of one’s professional self) and the external forces a teacher encounters in the achievement of teacher agency, which in turn resources one’s identity development as demonstrated above in this section.

The socio-cultural approach has been seen as being narrow in understanding learning, as over-emphasizing social and contextual influences, thus minimizing individual processes (Eteläpelto et al., 2013; Hökkä and Etelapelto, 2014). This study has employed a broader view of the socio-cultural approach that acknowledges the importance of working subjects (e.g., teachers) and the role of individual agency as well as that of sociocultural forces (Eteläpelto et al., 2013). This subject-centered lens provides conceptual space for exploring the complexity of multiple facets of a teacher’s professional self, as demonstrated in the teacher’s story, which complements Mezirow’s limitation in its humanistic view of seeing self as being unitary. This study thus argues that this holistic conceptual framework that integrates Mezirow’s theory and the subject-centered socio-culturally based approach to professional agency provides a conceptual direction for the research of transformative learning and teacher identity transformation.

## Conclusion

This study is a retrospective narrative case study based on a native CFL teacher’s lived experience across Chinese and Australian educational landscapes through an international teacher education program. It found that experiences leading to a disorienting dilemma, such as cross-cultural teaching experience, can trigger teachers to practice critical reflection. This should be incorporated into the design of an identity-oriented L2 teacher education program. It is also found that enacting new critical insights may further validate these insights and initiate a new cycle of critical reflection. Reflective journals may work as a method to encourage teachers to keep track of their own trajectory of thinking and encourage them to enact the revised insights. Further, teachers’ participation in teaching work and the school community may resource critical reflection and can advance teachers’ action in implementing new insights. Thus, how to enhance teacher agency counts for the various sides of an identity-oriented L2 teacher education program.

Out of the findings of this study, teacher agency has emerged as another vital element for implementing identity-oriented L2 teacher education practices. External conditions such as workload, institutional discourses, power relations and school culture can either constrain or resource teachers’ interest and enthusiasm for teaching. This constitutes critical knowledge for all parties involved in L2 teacher education programs to motivate teachers’ participation. Moreover, the complexity of dynamic interplay between individual and socio-cultural forces in the shaping of teacher agency indicates that teacher educators and policy makers should treat L2 teachers as whole persons with varying individual conditions and undergoing unique experiences within the same program. This suggests the importance of considering the ingredient of individuation (e.g., recognizing individual teachers’ prior knowledge) in the design of pedagogical practices focusing on teacher identity development.

For future investigation of teachers’ identity transformation researchers may consider including more CFL teachers with varying educational backgrounds in different social and cultural settings. It is also advisable to undertake a longitudinal approach in the future as it may provide more opportunities to capture more dynamic nuances. Further, teacher emotion has gained increasing recognition as a research lens in L2 teacher education (De Costa et al., 2018) and considering the dimension of emotion in future studies on CFL teacher identity transformation will generate new insights into L2 teacher education practices.

## Data availability statement

The original contributions presented in the study are included in the article/supplementary material, further inquiries can be directed to the corresponding author.

## Ethics statement

The studies involving human participants were reviewed and approved by Western Sydney University Ethics Committee. The patients/participants provided their written informed consent to participate in this study.

## Author contributions

SY conceived and designed the study, collected and analyzed the data, and wrote the manuscript. JH contributed to the analysis of the data and offered suggestions for revision. Both authors contributed to the article and approved the submitted version.

## Footnotes

1 Teacher identity in this article refers to a teacher’s teaching self, a facet of a teacher’s professional self.
